# Supplementary material for: Optical inactivation of intracellular molecules by fast-maturating photosensitizing fluorescence protein, HyperNova
Source: Commun Biol. 2024 Aug 6;7:945. doi: 10.1038/s42003-024-06583-x (PMC11303530; doi:10.1038/s42003-024-06583-x)
Supplement: Supplementary file 2 — Supplementary Information [file 42003_2024_6583_MOESM2_ESM.pdf]

Supplementary Information

Optical inactivation of intracellular molecules by fast-maturating photosensitizing fluorescence protein, HyperNova.

Hisashi Shidara, Taku Shirai, Ryohei Ozaki-Noma, Susumu Jitsuki, Takeharu Nagai and Kiwamu Takemoto*

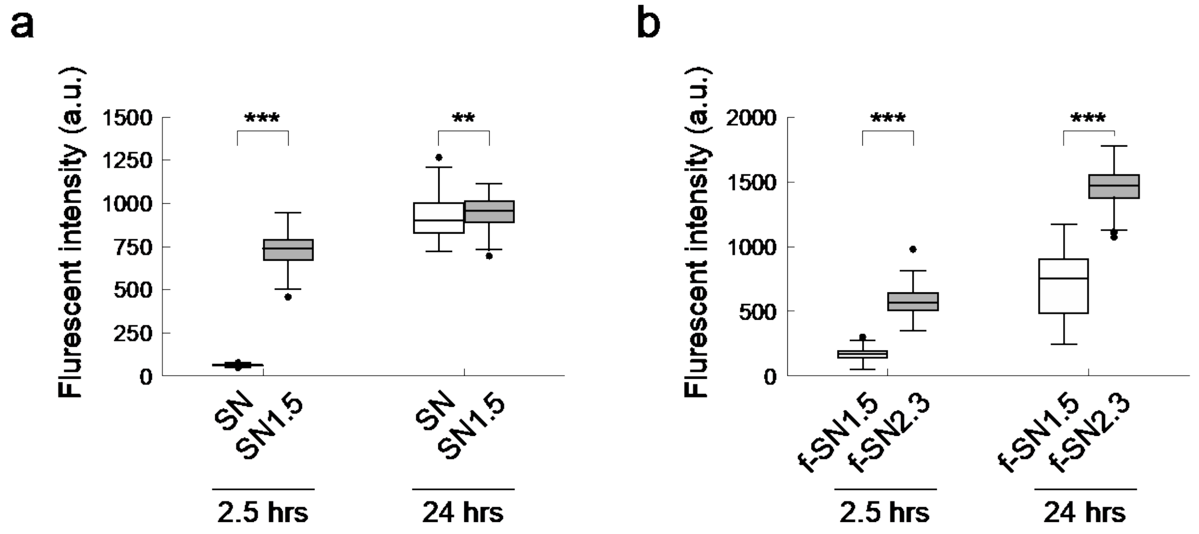

Supplementary Figure 1 | Mutagenesis screening of SuperNova variants to improve maturation efficiency at 37°C.

a Comparison of maturation efficiency in *E. coli* expressing SuperNova (SN) or SuperNova1.5 (SN-1.5) at 37°C 2.5 hours or 24 hours after IPTG induction. The average fluorescence intensity in colonies expressing SuperNova or SuperNova1.5 is shown (n = 150 colonies each). $p < 0.001$ in 2.5 hours and $p < 0.01$ in 24 hours by Wilcoxon rank sum test.

b Comparison of maturation efficiency in *E. coli* expressing ferritin fusion proteins at 37°C 2.5 hours or 24 hours after IPTG induction. The average fluorescence intensity in colonies expressing ferritin-SuperNova1.5 (f-SN1.5) or ferritin-SuperNova 2.3 (f-SN2.3) is shown. (n = 150 colonies each). $p < 0.001$ in 2.5 hours and $p < 0.001$ in 24 hours by Wilcoxon rank sum test.

**$p < 0.01$ and ***$p < 0.001$, significant difference.

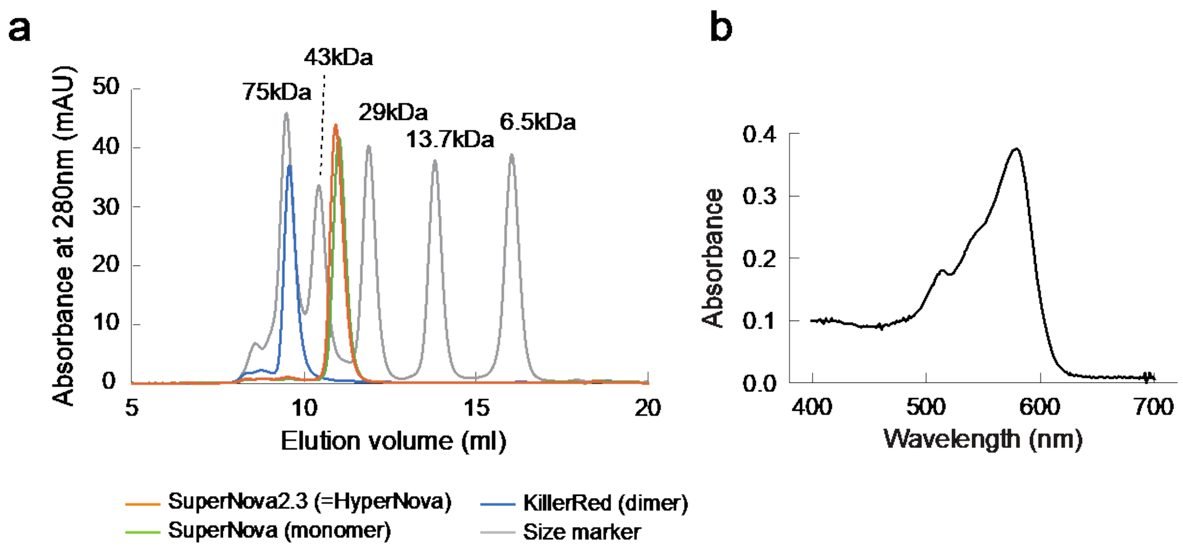

Supplementary Figure 2 | Spectroscopic analysis of HyperNova.

a Gel filtration analysis of HyperNova (orange line), KillerRed (blue line) and SuperNova (green line) and molecular weight marker proteins (gray line). Data are plotted as the measured absorption at 280 nm versus elution volume.

b Absorption spectra of HyperNova.

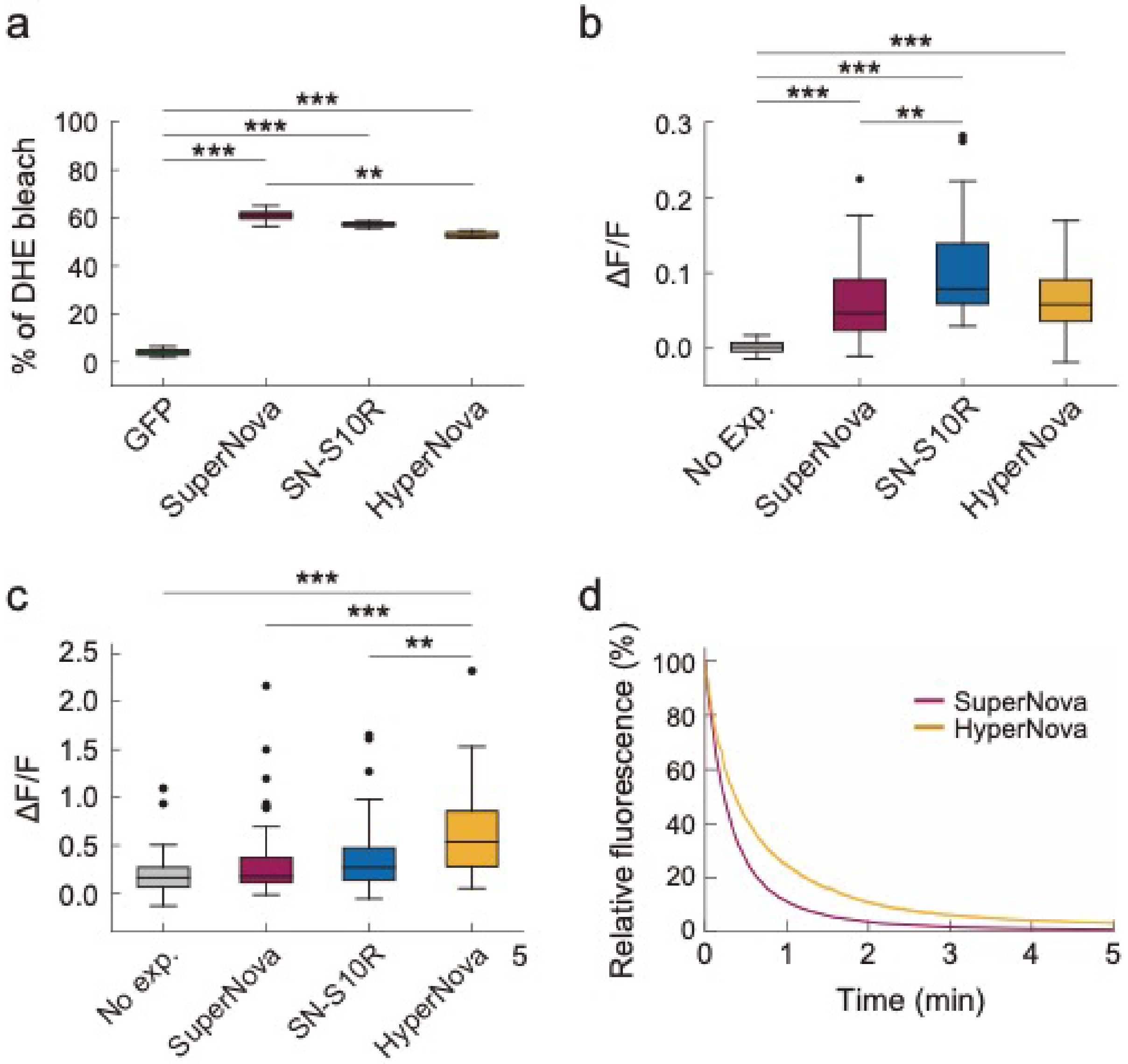

Supplementary Figure 3 | Comparative analysis of ROS generation and CALI efficiency in SuperNova variants.

a The superoxide production of HyperNova is rather slightly lower than that of SuperNova. *In vitro* comparison of superoxide generation in purified proteins by DHE indicator. The average reduction (%) in the fluorescence intensity of DHE by light irradiation is shown (n = 4). $p < 0.001$ in EGFP vs. SuperNova, EGFP vs. SN-S10R and EGFP vs. HyperNova, $p = 0.229$ in SuperNova vs. SN-S10R, $p < 0.01$ in SuperNova vs. HyperNova and $p = 0.128$ in SN-S10R vs. HyperNova by unpaired t test with Bonferroni correction. Note proteins well matured in *E. coli* at 23°C for 6 days were purified and used.

b Superoxide generation in HyperNova similar to that of SuperNova in living mammalian cells. The average increase in MitoSOX fluorescence intensity before and after light irradiation is shown (n = 35 cells for No exp., n = 41 cells for SuperNova, n = 32 cells for SN-S10R and n = 30 cells for HyperNova). $p < 0.001$ in NoExp. vs. SuperNova, NoExp.

vs. SN-S10R and NoExp. vs. HyperNova, $p < 0.01$ in SuperNova vs. SN-S10R, $p = 1.000$ in SuperNova vs. HyperNova and $p = 0.114$ in SN-S10R vs. HyperNova by Wilcoxon rank sum test with Bonferroni correction.

c Improved total ROS production in HyperNova. Total ROS production in living mammalian cells expressing SuperNova variants. The average fluorescence increase in the CellROX indicator after versus before light irradiation was compared (n = 43 cells for NoExp, n = 50 cells for SuperNova, n = 53 cells for SN-S10R and n = 47 cells for HyperNova). $p = 0.993$ in NoExp. vs. SuperNova, $p = 0.089$ in NoExp vs. SN-S10R, $p < 0.001$ in NoExp. vs. HyperNova, $p = 1.000$ in SuperNova vs. SN-S10R, $p < 0.001$ in SuperNova vs. HyperNova and $p < 0.01$ in SN-S10R vs. HyperNova by Wilcoxon rank sum test with Bonferroni correction.

d Photobleaching of HyperNova and SuperNova. The average reduction (%) of red fluorescence in response to intense light exposure was detected in HeLa cells expressing SuperNova or HyperNova (n = 15 cells in SuperNova and n = 13 cells in HyperNova).

$p < 0.01$ and ***$p < 0.001$, significant difference. Data points in **c are shown as the mean±s.e.m.

a

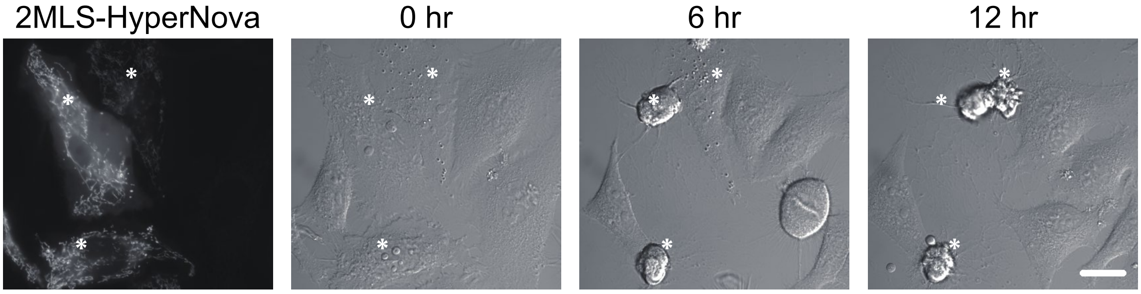

b

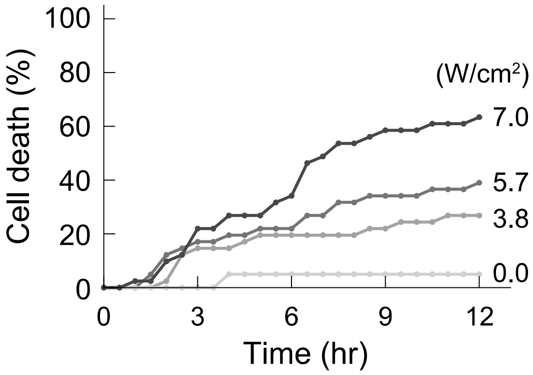

Supplementary Figure 4 | Cell death induction by CALI in mammalian cells overexpressing HyperNova in mitochondria.

a Images of fluorescence and DIC in HeLa cells expressing HyperNova in mitochondria examined in Fig. 1d. 2MLS indicates tandem mitochondria localization signal. Asterisk indicates cells expressing 2MLS-HyperNova. Bar indicates 20 μm. Note that because HyperNova matures rapidly, cytoplasmic leakage of its fluorescence is weakly observed, as shown in a previous report.[1]

b CALI efficiency of HyperNova in various light conditions. Cells expressing 2MLS-HyperNova were irradiated by the indicated light conditions. Data were plotted as the percentage of living cells versus time after light exposure (n = 41 cells for 7.0 W/cm^2 90 sec, 5.7 W/cm^2 90 sec and 3.8 W/cm^2 90 sec and n = 40 cells for 0.0 W/cm^2).

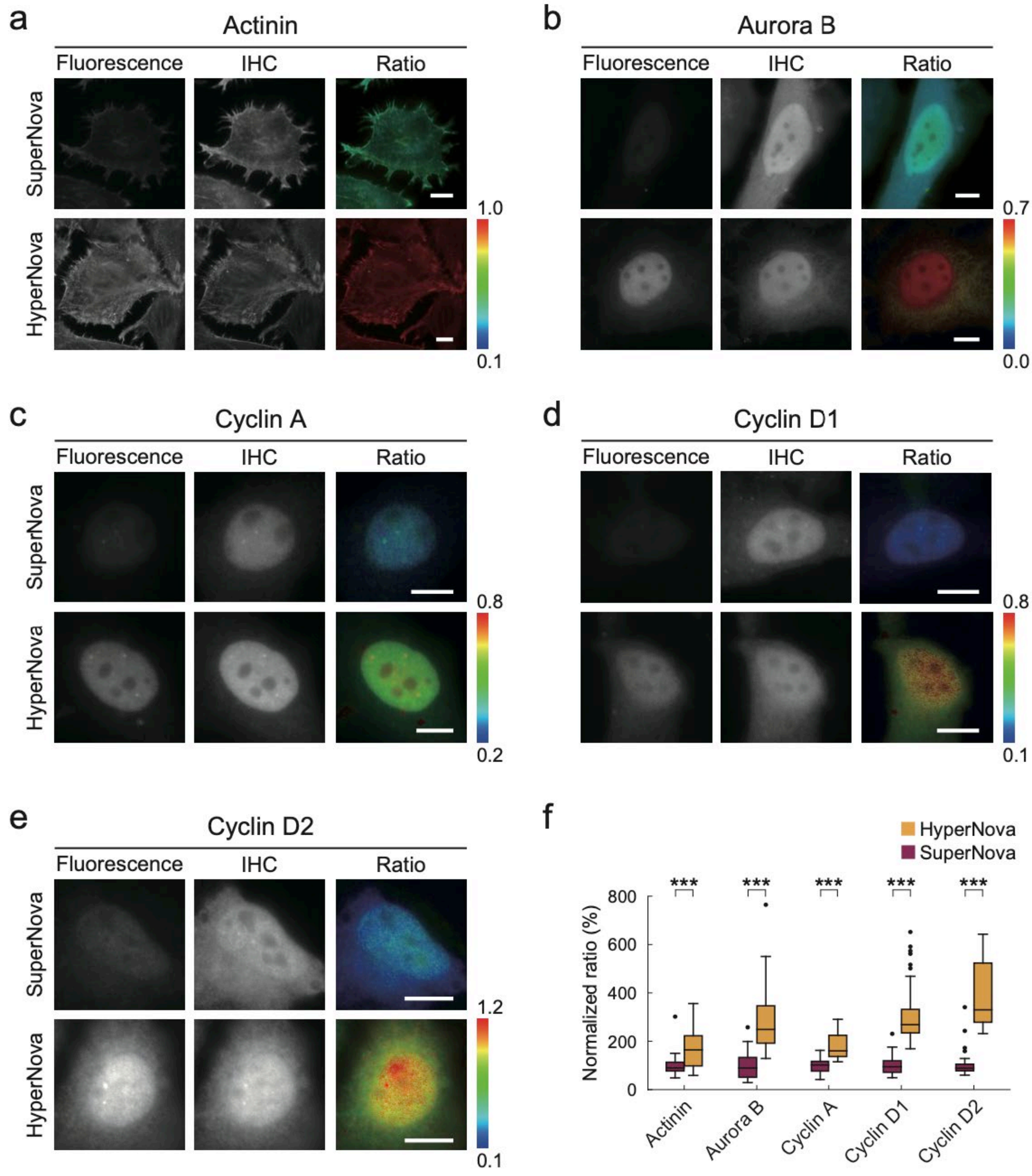

Supplementary Figure 5 | Enhanced maturation efficiency of HyperNova in fusion proteins.

a-e HeLa cells expressing Actinin (**a**), Aurora B (**b**), Cyclin A (**c**), Cyclin D1 (**d**) and Cyclin D2 (**e**) fusion proteins were subjected to immunostaining with an anti-KillerRed antibody and Alexa488-conjugated secondary antibody. The ratio image was calculated by fluorescence of SuperNova or HyperNova per that of Alexa488 to detect fluorescence

intensity per recombinant protein molecule. Their ratio images are shown in pseudocolor. IHC indicates immunohistochemistry. Bar indicates 10 μm.

f The average ratio of images expressing fluorescence per recombinant protein molecule is shown (n = 52 cells for Actinin-HyperNova, n = 48 cells for Actinin-SuperNova, n = 52 cells for Aurora B-HyperNova, n = 44 cells for Aurora B-SuperNova, n = 54 cells for Cyclin A-HyperNova, n = 48 cells for Cyclin A-SuperNova, n = 57 cells for Cyclin D1-HyperNova, n = 53 cells for Cyclin D1-SuperNova, n = 54 cells for Cyclin D2-HyperNova and n = 52 cells for CyclinD2-SuperNova). $p < 0.001$ for Actinin, Aurora B, Cyclin A, Cyclin D1 and Cyclin D2 by the Wilcoxon rank sum test.

***$p < 0.001$, significant difference.

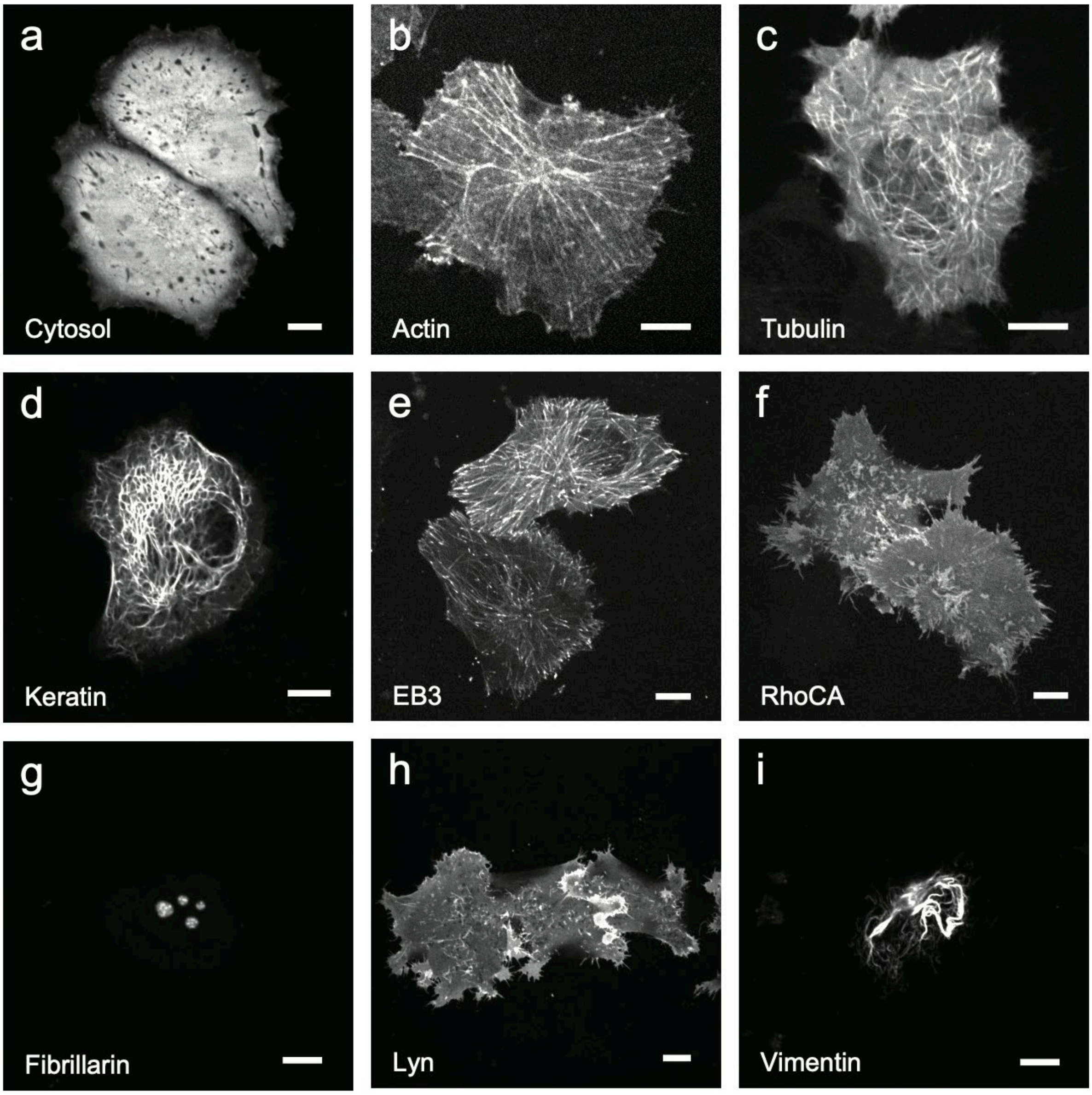

Supplementary Figure 6 | HyperNova fusion proteins expressed in living HeLa cells.

a HyperNova, a nonfusion protein that is evenly expressed in the cytosol and nucleus without any aggregation.

b Actin-HyperNova, highlighting a cytosolic actin fiber.

c Tubulin-HyperNova, highlighting a microtubule.

d Keratin-HyperNova, highlighting an intermediate filament.

e EB3-HyperNova. EB3 is a microtubule binding protein, so fusion proteins highlight microtubules.

f HyperNova-RhoCA. Rho-CA is a constitutively active Rho so that fusion proteins are localized in the plasma membrane.

g Fibrillarin-HyperNova. Fibrillarin is a ribonucleic protein so that fusion proteins localize in nucleoli.

h Lyn-HyperNova. Lyn is a signal sequence for plasma membrane localization, so the fusion protein highlights the plasma membrane.

i Vimentin-HyperNova, highlighting an intermediate filament.

The representative images were shown. Bar indicates 10 μm.

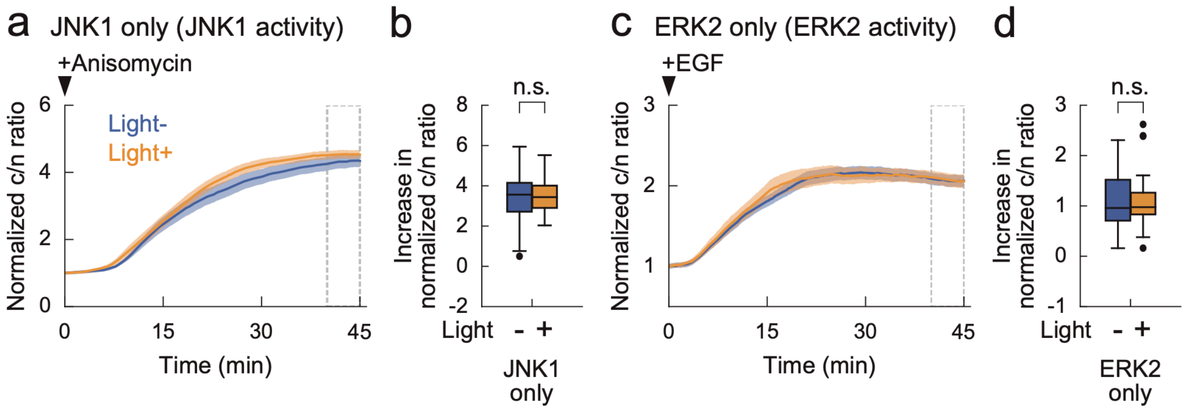

Supplementary Figure 7 | No effect on JNK1 and ERK2 activity after light irradiation without HyperNova.

a Control experiments without HyperNova in individual cells expressing JNK-KTR-GFP and JNK1 (n = 46 cells for light- and n = 43 cells for light+).

b Average increase in JNK-KTR-GFP signals from 40 to 45 min (dotted boxes in **A**) compared to the signals at 0 min. p = 0.357 in JNK1 with JNK-KTR-GFP by unpaired t test.

c Control experiments without HyperNova in individual cells expressing ERK-KTR-GFP and ERK2. (n=43 cells for light- and n= 44 cells for light+).

d Average increase in ERK-KTR-GFP signals at 40 to 45 min (dotted boxes in **C**) compared to the signals at 0 min. p = 0.929 in ERK2 with ERK-KTR-GFP by Wilcoxon rank sum test.

n.s. indicates not significant.

Supplemental Reference

1. Kashiwagi, S., Fujioka, Y., Satoh, A.O., Yoshida, A., Fujioka, M., Nepal, P., Tsuzuki, A., Aoki, O., Paudel, S., Sasajima, H., and Ohba, Y. (2019). Folding Latency of Fluorescent Proteins Affects the Mitochondrial Localization of Fusion Proteins. Cell Struct Funct *44*, 183-194. 10.1247/csf.19028.
